# Targeting Glutaminase by Natural Compounds: Structure-Based Virtual Screening and Molecular Dynamics Simulation Approach to Suppress Cancer Progression

**DOI:** 10.3390/molecules27155042

**Published:** 2022-08-08

**Authors:** Shams Tabrez, Torki A. Zughaibi, Mehboob Hoque, Mohd Suhail, Mohammad Imran Khan, Azhar U. Khan

**Affiliations:** 1 King Fahd Medical Research Center, King Abdulaziz University, Jeddah 21589, Saudi Arabia; taalzughaibi@kau.edu.sa (T.A.Z.); suhaildbt@gmail.com (M.S.); 2 Department of Medical Laboratory Sciences, Faculty of Applied Medical Sciences, King Abdulaziz University, Jeddah 21589, Saudi Arabia; 3 Applied BioChemistry Lab, Department of Biological Sciences, Aliah University, Kolkata 700160, India; m_hoque@aliah.ac.in; 4 Department of Biochemistry, Faculty of Science, King Abdulaziz University, Jeddah 22254, Saudi Arabia; imrankhaniitr@gmail.com; 5 Department of Chemistry, School of Life and Basic Sciences, SIILAS Campus, Jaipur National University, Jaipur 302017, India; azhar.u.kh@gmail.com

**Keywords:** glutamine, cancer, glutaminase, natural compounds

## Abstract

Cancer cells change their glucose and glutamine (GLU) metabolism to obtain the energy required to continue growing. Glutaminase (GLS) plays a crucial role in promoting cell metabolism for cancer cell growth; targeting GLU metabolism by inhibiting GLS has attracted interest as a potential cancer management strategy. Herein, we employed a sequential screening of traditional Chinese medicine (TCM) database followed by drug-likeness and molecular dynamics simulations against the active site of GLS. We report 12 potent compounds after screening the TCM database against GLS, followed by a drug-likeness filter with Lipinski and Veber rule criteria. Among them, ZINC03978829 and ZINC32296657 were found to have higher binding energy (BE) values than the control compound 6-Diazo-5-Oxo-L-Norleucine, with BEs of −9.3 and −9.7 kcal/mol, respectively, compared to the BE of 6-Diazo-5-Oxo-L-Norleucine (−4.7 kcal/mol) with GLS. Molecular dynamics simulations were used to evaluate the results further, and a 100 ns MD simulation revealed that the hits form stable complexes with GLS and formed 2–5 hydrogen bond interactions. This study indicates that these hits might be employed as GLS inhibitors in the battle against cancer. However, more laboratory tests are a prerequisite to optimize them as GLS inhibitors.

## 1. Introduction

Cancer is the leading cause of mortality globally [1]. In the United States, approximately 2 million new cancer cases and 0.6 million cancer fatalities are expected in 2022, with 350 deaths a day from lung cancer, the biggest cause of cancer mortality [2]. Cellular metabolic reprogramming is a well-known feature of cancer to sustain the high demand for energy required for growth [3]. Cancer cells change their glucose and glutamine (GLU) metabolism to get the energy they need to keep growing. Enhanced GLU metabolism is a characteristic of cancer and is recognized as a major metabolic alteration in cancer cells [4]. Many cancers rely on GLU catabolism to generate metabolic intermediates that support bioenergetics and biosynthetic needs [5]. Glutaminase (GLS) is a mitochondrial enzyme that starts this process by converting GLU to glutamate, which is then used in a number of activities that help tumor cells grow and survive, including energy production, amino acid synthesis, and glutathione formation. GLS activity corresponds with poor clinical prognosis in patients with breast, colorectal, lung, liver, and brain tumors, and rapid-growing tumor cells have enhanced GLS mRNA levels as well as elevated GLS expression [4,6,7,8,9]. GLS inhibition by siRNA has antiproliferative effects on cancer cells [10]. There is currently no FDA-approved GLS inhibitor on the market, and CB-839 (telaglenastat) is the only GLS inhibitor tested for clinical trials [11].

Natural compounds with a wide chemical diversity have been studied intensively for their anticancer potential. Efforts have been undertaken in recent decades to identify new natural compounds from bacteria, plants, and other living species. Natural products are thought to have accounted for about 25% of all newly authorized anticancer medications between 1981 and 2019 [12,13]. Irinotecan, vincristine, etoposide, and paclitaxel are plant-derived anticancer medicines; actinomycin D and mitomycin C are bacteria-derived anticancer therapeutics; and bleomycin is a marine-derived anticancer medication [14].

Drug development and discovery is a difficult, slow, and expensive process, but it has been speeding up because of the development of computational tools and approaches. The use of computer-assisted drug design (CADD) tools in early investigations by prominent pharmaceutical corporations and research organizations have aided in the acceleration of the drug discovery process while reducing expenses and failures in the final stage [15]. The use of rational drug design as a component of CADD gives helpful insights into the binding affinity and molecular interaction between target protein and ligand. In addition, supercomputing facilities, advanced programs, algorithms, and tools have aided lead discovery in pharmaceutical research [16]. This work aimed to identify new possible compounds from a traditional Chinese medicine (TCM) database using in silico methodologies that might be employed as GLS inhibitors to combat cancer.

## 2. Methodology

### 2.1. Protein Preparation

The 3D structure of GLS was obtained from the Protein Data Bank (PDB ID: 4O7D). It is a homo 4-mer structure, and we selected a monomer unit of GLS and minimized it for further screening.

### 2.2. Compound Library Preparation

The natural compound library comprising about 60 thousand compounds was downloaded from the TCM database. To prepare ligands (energy minimization and force field), the ‘Prepare Ligands module’ in Discovery Studio [17] 2020 was used. The control compound 6-Diazo-5-Oxo-L-Norleucine (DON; CID: 9087) was retrieved from the PubChem database.

### 2.3. Virtual Screening (VS) and Molecular Docking

VS plays an important role in the identification and development of new drugs [18]. Molecular docking-based VS is based on interacting receptors and small compounds. AutoDock Vina 1.1.2 and AutoDock 4.2.5.1 [19] were used for VS and in-depth docking studies. Top leads were retrieved based on the binding energy (BE) toward GLS.

### 2.4. Physiochemical and Drug-Likeness Properties Prediction

To estimate the physiochemical and drug-likeness properties, the top 20 compounds were selected from the VS results. The general physicochemical and drug-likeness properties of these compounds were analyzed using datawarrior tools [20], and these compounds were then filtered using Lipinski and Veber rules.

### 2.5. Molecular Dynamics Simulation

GROMACS 5.1.2 [21] was used to perform MD simulations on GLS-DON (control), GLS-ZINC32296657, and GLS-ZINC03978829 at 300 K, with the GROMOS96 43a1 force-field [22]. The PRODRG server was used to produce the compound’s topology and force-field parameters [23]. Charges were manually corrected in the topology file, new compound atoms were added to the complex topology files, and all of the compounds’ attributes were included in the system topology. GLS-DON (control), GLS-ZINC32296657, and GLS-ZINC03978829 were immersed in a ‘cubic box’ of water molecules with an initial diameter of 8 nm using the ‘gmx editconf’ module for boundary conditions and the ‘gmx solvate’ module for solvation. The charges on GLS-DON and screened complexes were neutralized by adding Na^+^ and Cl^−^ ions to preserve neutrality and a physiological concentration using the gmx genion module (0.15 M). All graphical representations of the 3D models were created using PyMOL and VMD [24].

## 3. Results and Discussion

GLU metabolism has long been a focus of cancer research due to its role in stressed settings. Because of GLS’s critical involvement in promoting cell metabolism for tumor cell proliferation, targeting GLU metabolism via GLS inhibition has received a lot of interest as a possible therapeutic approach for cancer management [4]. This paper screened the TCM database of natural compounds against GLS. We chose the top 20 compounds to further investigate their physicochemical and drug-likeness properties (Table 1). These (20 compounds) were further filtered using Lipinski and Veber rules to narrow down the screening results, and therefore only 13 compounds, including control, passed the prerequisites (Table 2).

**Figure 1:**
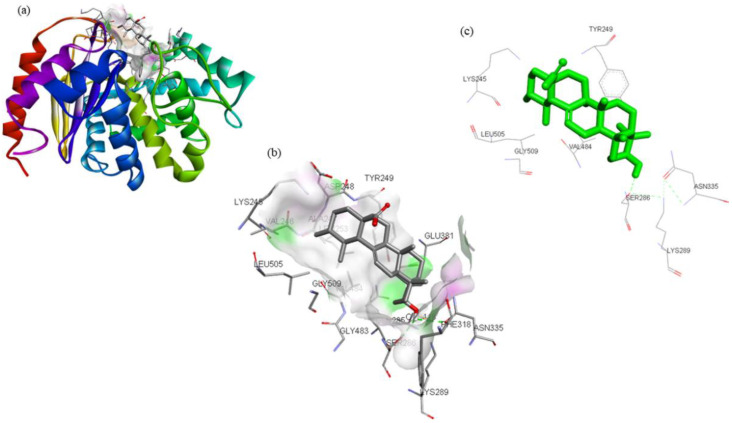
Visualization of ZINC03978829 (stick representation) in the binding pocket of GLS (**a**), interacting residues of GLS with ZINC03978829 (**b**,**c**).

**Figure 2:**
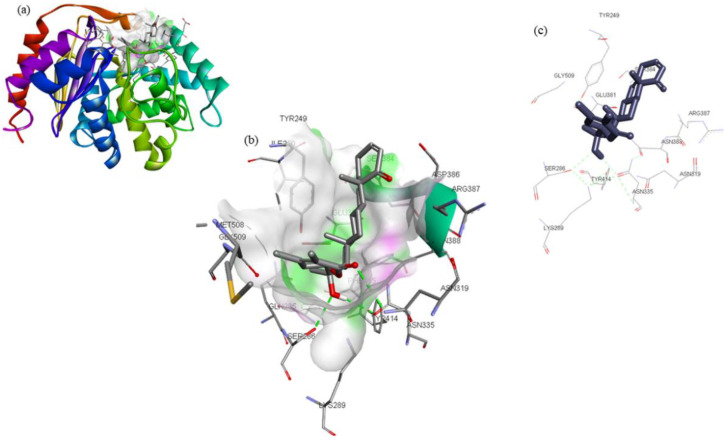
Visualization of ZINC32296657 (stick representation) in the binding pocket of GLS (**a**), interacting residues of GLS with ZINC32296657 (**b**,**c**).

By analyzing the binding poses of these 13 compounds with the GLS active site, it was determined that ZINC03978829 and ZINC32296657 have a strong affinity for GLS. ZINC03978829 was noted to interact with Lys245, Tyr249, Ser286, Lys289, Asn335, Val484, Leu505, and Gly509 residues of GLS (Figure 1), while Tyr249, Ser286, Lys289, Asn319, Asn335, Glu381, Ser384, Arg387, Asn388, Tyr414, and Gly509 residues of GLS were found to interact with ZINC32296657 (Figure 2). The BEs of ZINC03978829 and ZINC32296657 with GLS were noted to be −9.3 and −9.7 kcal/mol, respectively (Table 3).

**Figure 3:**
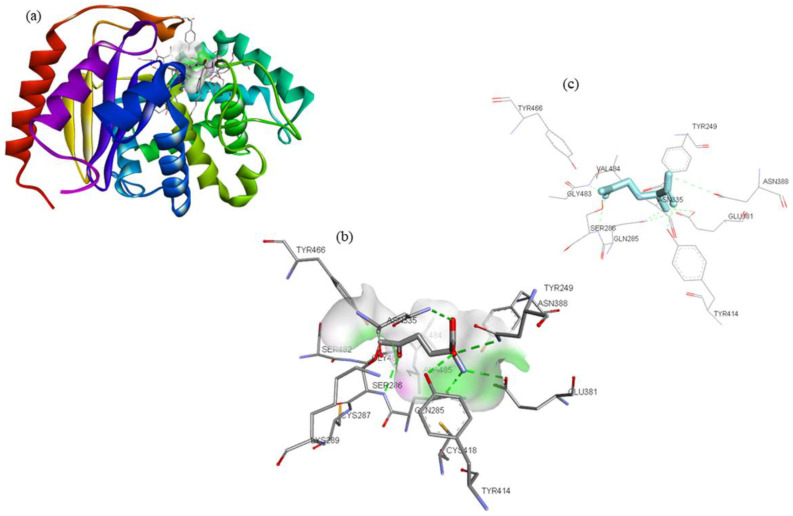
Visualization of DON (stick representation) in the binding pocket of GLS (**a**), interacting residues of GLS with DON (**b**,**c**).

DON, a glutamine analog, is a competitive inhibitor of GLS [25], and was used as the reference compound in the present study. DON was observed to interact with Tyr249, Gln285, Ser286, Asn335, Glu381, Asn388, Tyr414, Tyr466, Gly483, and Val484 residues of GLS (Figure 3). Tyr249, Ser286, and Asn335 were the common interacting residues of GLS with ZINC03978829, ZINC32296657, and the DON (Figure 1, Figure 2 and Figure 3). The BE of DON with GLS was noted to be −4.7 kcal/mol (Table 1).

A docked complex with a higher (negative) BE is often viewed as an effective protein–ligand interaction. As an outcome, the rate of dissociation of specific ligands from their binding targets would be slower, and their half-lives would be longer [26]. Interestingly, hits (ZINC03978829, ZINC32296657) exhibit higher BE with GLS than the DON (reference compound), indicating that these hits can be used as GLS inhibitors to fight cancer.

Bioinformatics plays an increasingly indispensable role in the integration of biological data as the diversity and complexity of data types have grown. For mining biologically active substances, there are now two types of data that are useful and accessible. The first is experimental biological activity data, such as anti-tuberculosis, anti-cancer, and anti-HIV biological activity data, which may be found in the PubChem database [27]. The second is curated data from multiple TCM databases regarding TCM plants and their derived compounds. Using bioinformatics to mine candidate molecules with diverse actions, the two forms of data present a new possibility [28]. TCM databases using the bioinformatics approaches have been used to identify inhibitors against many therapeutically important biological targets [17,29,30,31].

**Figure 4:**
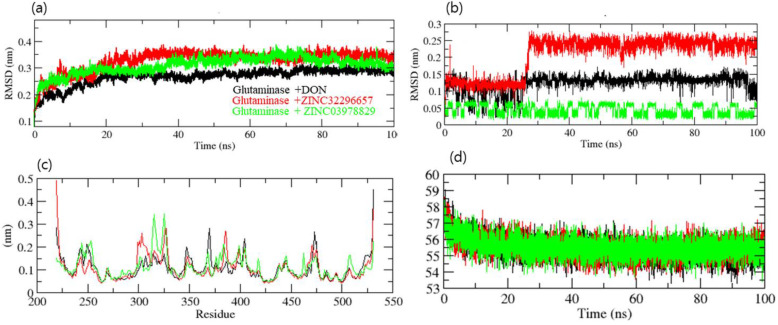
Dynamics study of GLS enzyme. (**a**) RMSD of the backbone of GLS, (**b**) RMSD of compounds, (**c**) RMSF, (**d**) volume and density of GLS.

The root mean square deviation (RMSD) is a protein stability metric; the lower the deviations, the more stable the protein structure [32]. GLS-DON (control), GLS-ZINC32296657, and GLS-ZINC03978829 had RMSD average values of 0.25, 0.33, and 0.31 nm, respectively. The RMSD plot revealed that GLU-ZINC03978829 binding increased GLS stability and resulted in fewer structural aberrations from its normal conformation. The bound structure of the GLS-ZINC32296657 complex is highly deviated (Figure 4a); it showed that the catalytic pocket of GLS enzyme was not forming a strong interaction with ZINC32296657, therefore it showed high deviation. In addition, the ligand RMSD also showed that DON and ZINC03978829 bind better than ZINC32296657 and are more stable (Figure 4b). Initially, ZINC32296657 formed a strong interaction with GLS until 22 ns, and after that the interaction started to lessen due to unstable bonding with the enzyme.

The GLS-ZINC32296657 and GLS-ZINC03978829 backbones displayed consistent fluctuations in the GLS catalytic pocket, most probably due to differing orientations, with the biggest fluctuation regions found between 300–325 and 370–400 residues (Figure 4c). The vibrations around the equilibrium are not random; rather, they are determined by the flexibility of the local structure. The average fluctuation of all residues throughout the simulation, as well as the root mean square fluctuation (RMSF) of GLS during binding of DON (control), ZINC32296657, and ZINC03978829, were plotted as a function of GLS residue numbers. The RMSF plot revealed that GLS has residual variants in various protein domain areas. Due to their close interaction with the GLS enzyme, DON and ZINC03978829 have been shown to reduce the residual fluctuations of the enzyme. Further, the volume and density plot revealed no significant differences between the hits and control compound (Figure 4d).

**Figure 5:**
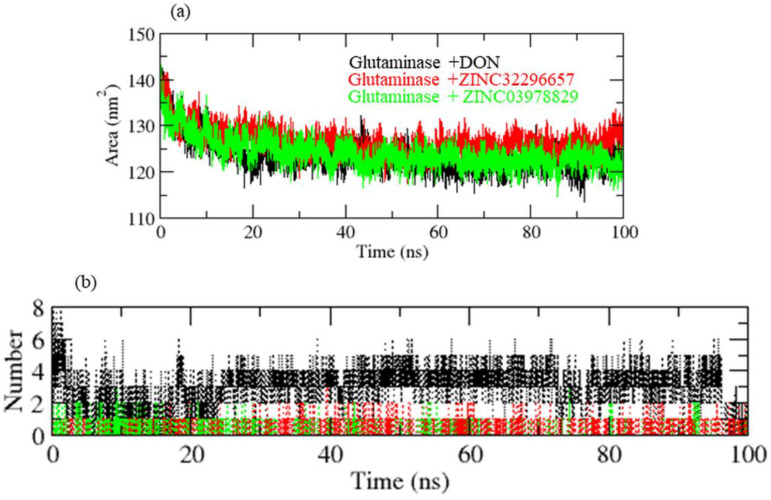
(**a**) SASA of GLS enzyme, (**b**) hydrogen bonding pattern of complexes.

A protein’s solvent-accessible surface area (SASA) is the area of its surface involved in the interaction with its solvent molecules. Average SASA values for GLS-DON (control), GLS-ZINC32296657, and GLS-ZINC03978829 complexes were recorded during the 100 ns MD trajectory. The SASA values for the GLS-DON (control), GLS-ZINC32296657, and GLS-ZINC03978829 complexes were 120.02, 134.01, and 125.30 nm^2^, respectively (Figure 5a). SASA analysis showed that upon binding of DON and ZINC03978829, surface exposure was reduced more than ZINC32296657.

The hydrogen bond is vital in the stability of the ligand–target complex [33]. Between protein and ligand, hydrogen bonds were formed within 0.35 nm. The stability of docked complexes was tested using 100 ns simulations of GLS-DON (control), GLS-ZINC32296657, and GLS-ZINC03978829 in a solvent environment. GLS-DON and GLS-ZINC03978829 form 2–5 hydrogen bonds with the GLS catalytic pocket, whereas GLS-ZINC32296657 forms 1–2 hydrogen bonds with the GLS catalytic pocket. GLS-ZINC32296657 formed fewer H-bonds due to instability in the pocket of the GLS enzyme (Figure 5b).

**Figure 6:**
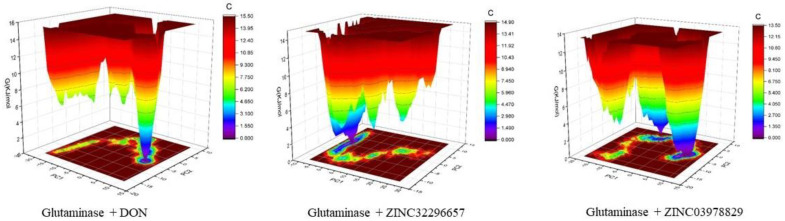
Gibbs free energy landscape of the complexes with GLS.

The energy landscape of the complexes GLS-ZINC32296657, GLS-ZINC03978829, and GLS-DON has been plotted. The blue color denotes the location with the least amount of energy. The GLS-ZINC03978829 complex has two distinct global energy minima basins (in blue), whereas the GLS-DON and GLS-ZINC32296657 complexes have comparable energy minima states. The blue spots represent greater stability, whereas more blue areas represent shifts in the protein structure followed by the thermodynamically more favorable state (Figure 6).

## 4. Conclusions

Considering GLS as a viable therapeutic target because of its role in cancer development, targeting it with the selected hits is an interesting approach. The in silico technique utilized in this work might be effective in identifying possible hits from natural compounds as potent GLS inhibitors. MD simulations indicated that the hit compounds (ZINC03978829 and ZINC32296657) strongly bind to GLS and are stable. This study implies that these hits from the TCM database could be used as GLS inhibitors to fight cancer. It is noteworthy that the BE estimates and MD simulations only illustrate the interaction efficiency and stability of hits with GLS; further experimental studies are a prerequisite to optimize these hits (ZINC03978829 and ZINC32296657) as GLS inhibitors.

## Figures and Tables

**Table 1:** Top 20 screened compounds with their physicochemical and drug-likeness properties.

Compound Name	Mol. wt	cLogP	cLogS	H-Acceptors	H-Donors	Drug-Likeness	Mutagenic	Tumorigenic	Reproductive Effective	Irritant	Drug Score
ZINC42835355	608.733	6.1432	−6.513	8	2	4.6261	none	none	none	none	0.221249
ZINC17465983	546.526	4.8554	−7.85	10	6	1.0735	none	high	low	none	0.115695
ZINC05823171	436.59	4.4475	−4.742	4	2	0.4587	none	none	none	none	0.433376
ZINC05618656	470.476	4.4782	−4.586	7	6	−0.47845	none	none	high	none	0.214637
ZINC38800324	436.59	5.3389	−6.055	4	2	−0.61958	none	none	none	none	0.252781
ZINC28642721	538.463	2.9812	−6.422	10	6	−0.73675	none	none	none	none	0.257962
ZINC13384046	484.459	4.3143	−5.366	8	7	−0.75118	high	high	none	none	0.107838
ZINC33830992	442.725	6.499	−6.347	2	2	−0.95276	none	none	none	none	0.189819
ZINC32296657	438.606	4.4297	−5.029	4	1	−1.0323	none	none	none	none	0.323614
ZINC05520028	538.463	4.68	−8.842	10	6	−1.1275	none	none	none	high	0.104357
ZINC34124041	470.779	7.9297	−7.327	2	0	−3.2521	none	none	none	none	0.117694
ZINC04097720	426.726	7.5888	−6.968	1	0	−3.3053	none	none	none	none	0.132645
ZINC04722964	426.726	7.5888	−6.968	1	0	−3.3053	none	none	none	none	0.132645
ZINC08214547	576.768	3.0443	−5.279	8	4	−3.6238	none	none	none	none	0.221118
ZINC03978829	456.708	6.0021	−6.111	3	2	−3.658	none	none	none	none	0.164992
ZINC33831792	422.694	7.3798	−6.44	1	0	−4.1072	none	none	none	none	0.140914
ZINC31165761	424.71	7.4843	−6.704	1	0	−4.5197	none	none	none	none	0.134382
ZINC06041333	632.879	7.6697	−7.838	6	2	−6.334	none	none	none	none	0.082226
ZINC33831775	424.71	7.4221	−6.687	1	0	−6.4	none	none	none	none	0.133989
ZINC05884271	514.441	4.4706	−7.734	10	6	−6.6779	none	high	none	none	0.094558
DON	145.157	−2.6809	−0.868	4	2	−19.893	none	none	none	high	0.295486

**Table 2:** List of compounds filtered by Lipinski and Veber rules.

Name	Mol. Wt.	ALogP	Rotatable Bonds	Molecular_Polar Surface Area
ZINC31165761	424.702	7.404	0	17.07
ZINC32296657	438.599	5.328	2	63.6
ZINC33830992	442.717	6.451	0	40.46
ZINC33831775	424.702	7.449	0	17.07
ZINC33831792	424.702	7.598	0	17.07
ZINC34124041	470.77	7.865	2	26.3
ZINC38800324	436.583	5.522	0	74.6
ZINC03978829	455.692	5.018	1	60.36
ZINC04097720	426.717	7.443	0	17.07
ZINC04722964	426.717	7.443	0	17.07
ZINC05823171	436.583	4.261	0	74.6
ZINC08214547	576.761	2.887	3	117.84
DON	144.149	−2.354	4	83.22

**Table 3:** BE and interacting residues of hits with GLS.

Compounds	Structure	Target	Binding Energy (kcal/mol)	Interacting Residues
ZINC03978829	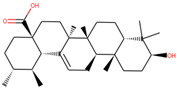	GLS	−9.3	Lys245, Tyr249, Ser286, Lys289, Asn335, Val484, Leu505, and Gly509
ZINC32296657	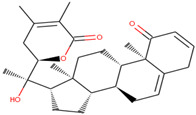		−9.7	Tyr249, Ser286, Lys289, Asn319, Asn335, Glu381, Ser384, Arg387, Asn388, Tyr414, and Gly509
DON *	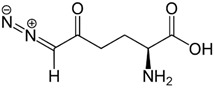		−4.7	Tyr249, Gln285, Ser286, Asn335, Glu381, Asn388, Tyr414, Tyr466, Gly483, and Val484

* Control compound.

## Data Availability

Data will be available upon request to the corresponding author.

## Abbreviations

GLU	Glutamine
GLS	Glutaminase
TCM	Traditional Chinese medicine
BE	Binding energy
MD	Molecular dynamics
CADD	Computer-assisted drug design
VS	Virtual screening
DON	6-Diazo-5-Oxo-L-Norleucine
RMSD	Root mean square deviation
RMSF	Root mean square fluctuation
SASA	Solvent-accessible surface area
