# Correction: Tabrez et al. Targeting Glutaminase by Natural Compounds: Structure-Based Virtual Screening and Molecular Dynamics Simulation Approach to Suppress Cancer Progression. *Molecules* 2022, *27*, 5042

**DOI:** 10.3390/molecules28010104

**Published:** 2022-12-23

**Authors:** Shams Tabrez, Torki A. Zughaibi, Mehboob Hoque, Mohd Suhail, Mohammad Imran Khan, Azhar U. Khan

**Affiliations:** 1 King Fahd Medical Research Center, King Abdulaziz University, Jeddah 21589, Saudi Arabia; 2 Department of Medical Laboratory Sciences, Faculty of Applied Medical Sciences, King Abdulaziz University, Jeddah 21589, Saudi Arabia; 3 Applied BioChemistry Lab, Department of Biological Sciences, Aliah University, Kolkata 700160, India; 4 Department of Biochemistry, Faculty of Science, King Abdulaziz University, Jeddah 22254, Saudi Arabia; 5 Department of Chemistry, School of Life and Basic Sciences, SIILAS Campus, Jaipur National University, Jaipur 302017, India

Due to miscommunication, in the original publication there is a discrepancy in the project number and funding institution, located in Funding Information and Acknowledgement [1]. The correct funding information should be as follows:

**Original:**

Funding: Deputyship for Research & Innovation, Ministry of Education in Saudi Arabia [Grant number IFPRC-146-141-2020].

**This should be replaced with:**

Funding: This research work was funded by Institutional Fund Projects under grant No. (IFPDP-39-22).

The authors state that the scientific conclusions are unaffected. All co-authors agree with the content of this correction and wish to apologize for any inconvenience caused for the readers resulting from this error.
